# Tissue expression of antigens of ABH blood groups in species of New World Monkeys *(Aotus infulatus*, *Callithrix jacchus*, *Sapajus apella and Saimiri sciureus)*

**DOI:** 10.1371/journal.pone.0241487

**Published:** 2020-11-18

**Authors:** Délia Cristina Figueira Aguiar, Washington Luiz Assunção Pereira, Gyselly de Cássia Bastos de Matos, Klena Sarges Marruaz da Silva, Rosane do Socorro Pompeu de Loiola, Tereza Cristina Oliveira Corvelo

**Affiliations:** 1 Laboratory of Biomolecular Technology, Institute of Biological Sciences, Federal -University of Pará, Belém, Pará, Brazil; 2 Pathology Laboratory, Rural Federal University of Amazonia, Belém, Pará, Brazil; 3 Institute of Health and Animal Production, Veterinary Medicine Course, Rural Federal University of Amazonia, Belém, Pará, Brazil; 4 Institute of Science and Technology in Biomodels, Oswaldo Cruz Foundation, Rio de Janeiro, Rio de Janeiro, Brazil; 5 Epidemiology Division, Pará State Health Secretary, Belém, Pará, Brazil; 6 Laboratory of Immunogenetics, Institute of Biological Sciences, Federal University of Pará, Belém, Pará, Brazil; Sichuan University, CHINA

## Abstract

ABH antigens are histo-antigens, but were first described on the surface of human erythrocytes. They are found in those cells only in great apes and humans, while in more primitive animals they are found in tissues and body fluids. ABH antigens are mainly distributed in tissues that are in contact with the external environment and may serve as ligands for pathogens in tissues or block their connection. Description of the distribution of these molecules in non-human primate tissues is restricted to a few tissues and species. This paper describes the expression of human A, B and H type antigens in different organs from four species of New World Primates, obtained from the Centro Nacional de Primatas, as well as comparing that expression with what has been described for humans. In this study, although the tissue description of the antigens is similar to the genetic model for humans, some differences in expression between some organs from those species and those of humans were found. The differences occurred mainly in endodermal organs that have secretory functions and are probably under the control of the human-type FUT-2 enzyme. In the mesodermal-origin organs there was a reduction or absence of A and B antigen marking, particularly in the H precursor substance, indicating that those organs are under the control of the human-type FUT-1 enzyme. These findings have demonstrated that there is similar ABH antigen reactivity in tissue distribution between the species, although there are some species-specific cases.

## Introduction

Antigens of the A, B and O blood groups are carbohydrate structures of glycoproteins and glycolipids. Several polymorphic genes are involved in regulating the synthesis of these glycoconjugates. The steps for their biosynthesis and chemical structure are explained by the relation between the ABO, H and Se systems [1].

ABO and H antigens may be formed from at least four types of precursor chains. In most tissues, the stages of biosynthesis of ABH structures are correlated with the embryological development of the tissue and its cellular differentiation. The expression of those varies from cell to cell and organ and organ [2], and the detection and location of ABH structures in various normal tissues in humans has been described in many studies [3]. The presence of ABH antigens was described initially on the surface of erythrocytes, although those are primarily histo-antigens. The distribution of those structures is extremely varied; from an evolutionary point of view they occur in tissues and fluids of more primitive mammalian species and appear in red blood cells only among the great apes and humans [4, 5].

Three α-2-fucosyltransferase genes (*FUT1*, *FUT2*, *Sec1*) has been characterized in primates [6]. They shared a high degree of DNA sequence identity, suggesting that they were generated by successive gene duplications and divergent evolution. In the course of evolution, the duplication event at the origin of *H* and *Se* genes occurred before the great mammalian radiation [7]. Besides, it has been proposed that an ancestral *Se* gene has been duplicated in two related genes, *Se* and *Sec 1* [8]. Then *FUT1* regulates the expression of H antigens on red cells and vascular epithelium. The secretor (*Se*) *FUT2* regulates the expression of H antigen in the endoderm derived epithelium. There exists a third α-2-fucosyltransferase gene called *Sec1*, which is inactivation.

In human *Sec1* is a pseudogene but others nonsense inactivating mutation might have occurred in the *Sec1* gene families, which may be responsible for the changes present in different species of New World Monkeys, suggesting the non-functionality as found for *Callithrix*, *Aotus*, and *Pithecia* whereas *Sapajus* and *Saimiri* have an active state for Sec 1 [9].

In this context, Old World Monkeys (OWM) and New World Monkeys (NWM) express ABH substances in secretions and body fluids, whereas in their red blood cells have only a factor related to the human B factor known as “like-B” [10].

In the specific case of ABH antigens derived from type 1 and 2 precursor chains, the type 1 chain is expressed mainly in tissues originating endodermally, under control of the *Se* gene, and found in fluids and bodily secretions [11]. The type 2 chain is expressed mainly in tissues of mesodermal and ectodermal origin, under control of gene *H* [12].

Regarding the distribution of these molecules in non-human primates, the studies are restricted to a few tissues and a few species [13–16]. The purpose of the present study was to clarify the distribution of ABH antigen expressions in several tissues of different New World Monkeys species of the Amazon region.

## Material and methods

### Animals

Tissues from various organs of 19 New World primates of the species *Aotus infulatus* (n = 3), *Callithrix jacchus* (n = 6), *Sapajus apella* (n = 6) and *Saimiri sciureus* (n = 4) were obtained from the Centro Nacional de Primatas (Pará, Brazil) for this study.

In all CENP non-human primate colonies, adequate environmental and nutritional conditions are maintained for the well-being of the animals and reproducing them, either to use them in scientific research or with the conservation of species. To achieve these objectives, there are reproductive, health and nutritional management in animals. Also, laboratory tests, routine clinical and surgical care, diagnostic imaging tests, microbiological control of conservation and reproduction areas and *post mortem* tests are carried out to investigate the cause of death of animals found dead in cages, as well as those who were already being treated at the clinic.

The species studied were kept in indoor cages and received different types of environmental enrichment. All species received feeding enrichment daily with different food items such as larvae of mealworm (*Tenebrio molitor*), varied seasonal fruits and seeds, offered on alternate days. In the case of *C*. *jachus* and *A*. *infulatus* species, environmental enrichment with furniture and bedding material was also offered, such as nest boxes and pine bedding. Non-human primates received specific industrial animal feed (MegaZoo^®^) and were fed according to the management of each species adopted by the institution: different types of fruits, vegetables, roots, tubers, milk, eggs, vitamins, and mineral supplements into the water *ad libitum*.

No animals in this study were euthanized. Most of the primates used were found dead on the floor of the cages due to natural death or a fight, while the rest were being treated at the clinic for different causes, such as abortion, cage fights and also due to the callitrichid slimming syndrome. The ones found dead in the morning were placed in refrigeration for the autopsy procedure by the pathologist responsible for investigating the cause of death.

It is important to mention that both for samples of primates belonging to the collection of paraffinized tissues and for those obtained from animals that died during this research, none of them were participating in other scientific experiments, which was an exclusion criterion for due research. Through the analysis of the animals’ clinical files, necropsies, and the result of the histopathological analysis of the organs, Table 1 indicates the cause of death of each animal. Besides, another point that must be informed is that another work of this research has already been published, by the same research group, relating the expression of ABH antigens in the stomach and the association of infection by the bacterium *Helicobacter* sp. [12].

**Table 1:** Species used in the study, their medical records, identifications used in CENP and the causes of death of the animals.

Species	Medical Record	Tatoo	Chip	Cause of death (Clinical information, necropsy and histopatology)
*A*. *infulatus*	055/05	WT	WC	Chronic inflammatory nephropathy
*A*. *infulatus*	002/05	AH-AKR	WC	*Cardiogenic shock*
*A*. *infulatus*	057/07	WT	WC	Inconclusive (found dead on the floor of the cages due to natural death or a fight)
*C*. *jacchus*	018/05	AD-BAN	WC	Wasting marmoset syndrome
*C*. *jacchus*	180/05	AD-BBU	WC	Wasting marmoset syndrome
*C*. *jacchus*	190/05	AD-BBP	WC	Probable bacterial peritonitis
*C*. *jacchus*	178/05	AD-BAO	WC	Inconclusive (found dead on the floor of the cages due to natural death or a fight)
*C*. *jacchus*	191/05	AD-BBF	WC	Probable bacterial peritonitis
*C*. *jacchus*	136/05	WT	WC	Wasting marmoset syndrome and Stronguloides hyperinfection
*S*. *Sciureus*	030/04	WT	039.573.030	Inconclusive (found dead on the floor of the cages due to natural death or a fight)
*S*. *Sciureus*	037/04	AT-BAT	WC	Multidiversified parasitic infection
*S*. *Sciureus*	152/05	AT-AIJ	039.226.770	Inconclusive (found dead on the floor of the cages due to natural death or a fight)
*S*. *Sciureus*	163/05	AT-BBO	039.330.131	Inconclusive (found dead on the floor of the cages due to natural death or a fight)
*S*. *apella*	151/05	AM-ANY	039.535.581	Chronic interstitial lung disease
*S*. *apella*	001/05	AM-AOH	WC	*Cardiogenic shock*
*S*. *apella*	013.06	AM-AQO	039.554.085	Inconclusive (found dead on the floor of the cages due to natural death or a fight)
*S*. *apella*	011.06	AM-BAF	WC	Inconclusive (found dead on the floor of the cages due to natural death or a fight)
*S*. *apella*	044.06	AM-AJU	WC	Inconclusive (found dead on the floor of the cages due to natural death or a fight)
*S*. *apella*	042/04	AM-AJE	WC	Systemic parasitic infection by Strongyloides

WT = Without Tatoo; WC = Without Chip.

The number of organs of the animals available among the species for use in the study varied, since some were obtained from the collection of tissues paraffinized at o CENP in 2004, while the remainder were collected during post-mortem autopsies on the animals.

The samples collected during the autopsies were fixed in 10% buffered formaldehyde and processed in paraffin. Later, 5μm slices were made and placed on histological slides. Only tissues without any pathology were used in this study.

### Methods

The content of the manuscript is original and it has not been published or accepted for publication, either in whole or in part, in any form. No part of the manuscript is currently under consideration for publication elsewhere.

#### Ethics statement

This research was approved by the Environmental Agency (IBAMA N° 086/2004) and by the Research Ethics Committee of the Instituto Evandro Chagas (N° 069–2005).

#### Immunohistochemistry

To determine the expression of A, B, and H antigens in primate organs in CENP we used the modified immunoperoxidase technique of Pedal et al., 1989 [17]. Anti-A, anti-B (Fresenius) monoclonal antibodies were used at a 1:10 dilution, while for H antigen, we used the *Ulex europaeus* lectin (Sigma—UEA I) linked to the peroxidase at a (1:50) dilution. The tissue slices that had been processed beforehand were then deparaffinized in xylol and treated with methanol containing 0.3% of H_2_O_2_. Next, the sections were washed in phosphate buffer (pH 7.6) and incubated with a blocking solution (phosphate buffer and bovine albumin). This was followed by incubation with the monoclonal antibodies and the *U*. *europaeus* lectin for one hour at room temperature, followed by washing with the phosphate buffer. The slides incubated with the anti-A and anti-B antibodies were treated with a blocking solution followed by a second incubation with anti-mouse IgG linked to peroxidase for one hour. After washing in phosphate buffer, the reaction was developed for all of the antigens (Tris buffer, diaminobenzidine, and hydrogen peroxide). The slides were stained with hematoxylin, dried, and mounted with Entellan^®^ (Sigma).

Slides were evaluated under a light microscope with results expressed as a score based on the percentage of the total field staining positively based of the following semi-quantitative scale: (+++) > 50% of the field showing positive staining; (++) < 50% of the field showing heterogeneous positive staining.

#### Characterization of the animals’ blood group phenotype

The ABH phenotype of each animal was determined by the expression of these antigens in organs that produce fluids and secretions (stomach, intestines, bladder, and salivary glands) since all primates are secretors of ABH substances [6]. This type of determination has already been used in another study [15], where the saliva or other secretions from the animal were not available.

#### Statistical analysis

The intra- and inter-specificities of NWM were estimated based on the variable ABH expression of antigens by the semi-quantitative scale (score). The means, standard deviations, and Shannon-Wiener Index were calculated using Bioestat 5.0 software [18].

## Results

### Immunohistochemistry of the substances in the human-type ABO blood groups of NWM

All of the animals of the species *C*. *jacchus* and *A*. *infulatus* belonged to blood groups A and B, respectively. In the species *S*. *apella*, four animals had the phenotype for blood group A and two for phenotype B. In the four individuals for the species *S*. *sciureus* tested, two had phenotype A and the rest were phenotype AB. In all of the organs tested the ABH expressions were concordant amongst themselves, in relation to the ABO blood group phenotype.

### Immunohistochemical distribution of antigens of human-type ABH blood groups in different species of NWM

**Fig 1:**
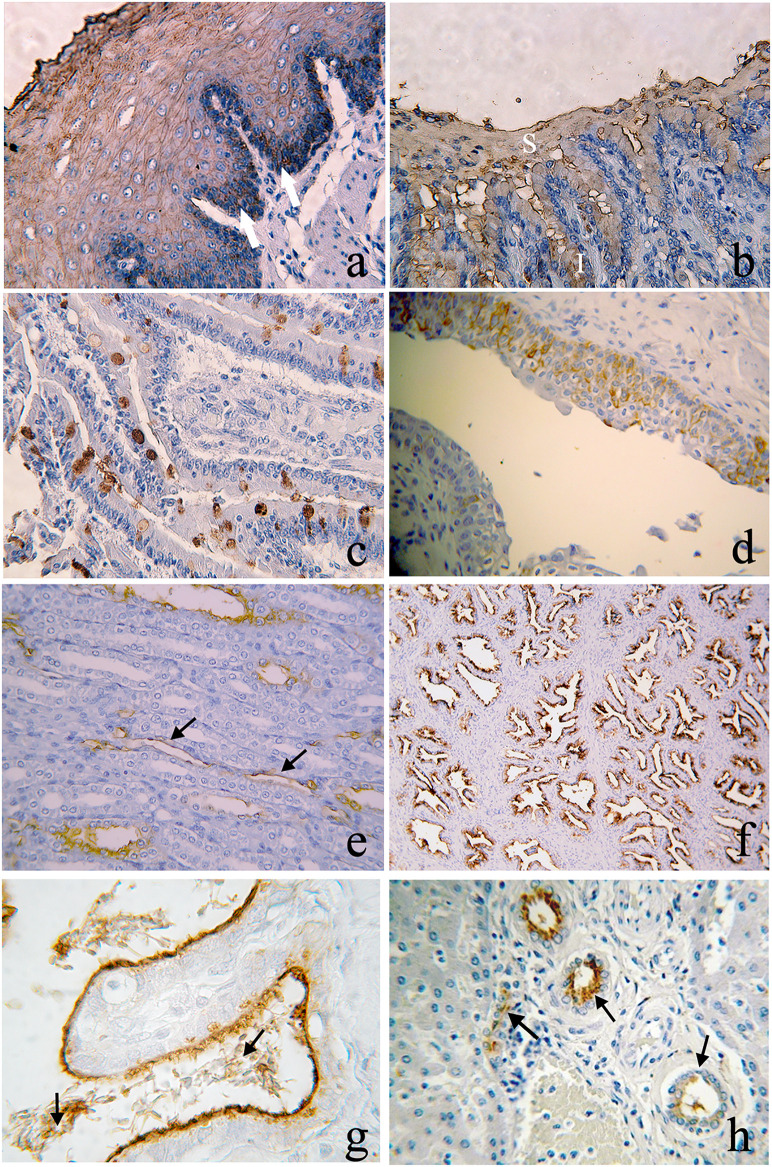
Expression of ABH antigens in *A*. *infulatus and C*. *Jacchus*. (a) anti-B in the basal layer (arrows) of the esophagus; (b) anti-H at the superficial (S) and intermediate (I) layers of the gastric mucosa; (c) anti-H in goblet cells of the small intestine; (d) anti-H in the bladder epithelium; (e) anti-A in the renal loop of Henle (arrows) - 400x; (g) anti-A epithelium in the prostatic glands. 100x; (h) anti-A in the epithelium (brown arrows) of the seminal vesicle and spermatozoids (black arrow). 1000x; (h) anti-H in the major and minor hepatic ducts (arrows) - 400x.

**Fig 2:**
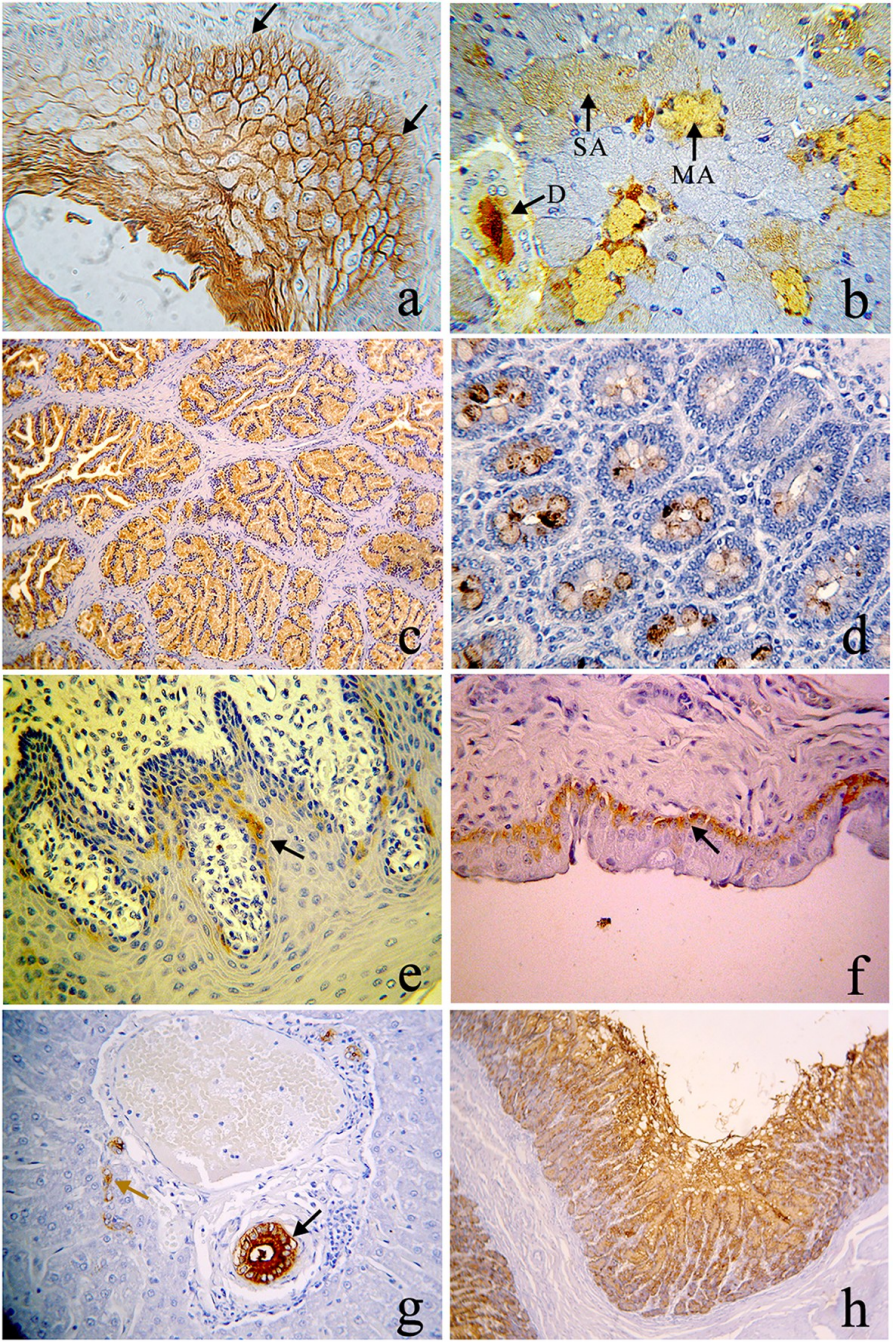
ABH antigens in *S*. *apella* and *S*. *sciureus* in organs with an endodermal origin. Pharynx (a) anti-H, including the basal (arrows), 400x; (b) anti-H in ducts (D), serous acini (SA), mucous (MA) of the salivary gland, 400x; (c) anti-A in the prostatic epithelium, 100x; (d) anti-B in goblet cells of the large intestine, 400x; (e) anti-H in the vaginal epithelium, 400x; (f) anti-H in the basal layer of the bladder (arrow), 400x; (g) Anti-B in the greater ducts (brown arrow) and lesser ducts (black of arrow) of the liver in *S*. *apella*, 400x; (h) anti-B in the gastric mucosa, 400x.

Only two of the organs studied (adrenal and heart) did not have expression of antigens from ABO blood groups. The expression found in the organs and tissues of each species analyzed are distributed according to the embryological origin of the tissue and detailed in Tables 2 and 3. Two patterns of antigen expression were observed in histological analyses. One pattern was homogeneous, with expression of the antigen in the entire area of tissue analyzed, and the other was heterogeneous, which areas completely marked as well as areas where the antigen did not occur. The antigen expressions in some of the tissue areas analyzed in the species studied are illustrated in Figs 1 and 2.

**Table 2:** Expression of ABH antigens in organs with an endodermal origin in species of New World Monkeys.

TISSUE Layer/ (n) cell type	Species/ABH Expression			
	*A*. *infulatus*	*C*. *jacchus*	*S*. *apella*	*S*. *sciureus*
ESOPHAGUS (n = 1; 2; 1; 1)				
Superficial epithelium	**BH**	**A/AH**	B**H**	AB
Suprabasal	**B**H	**A**/AH	**BH**	**AB**
Basal	**B**	A/SE	SE	B
PHARYNX (n = 2)	NT	NT		NT
Superficial epithelium	-	-	**A**H/**BH**	-
Suprabasal	-	-	**A**H/**H**	-
Basal	-	-	H	
STOMACH (n = 2; 3; 1; 3)				
Surface Layer	B/**B**H	AH*/AH	**BH**	**A**H/**ABH**/**AB**H
Intermediate	B/BH	AH*/AH	**B**H	**A**H/**AB**/**AB**H
Basal	B/B	AH*/AH	**B**H	**A/AB**/AB
SMALL INTESTINE (n = 2; 1; 3; 3; 2)				
Absorbent Cells	**B**	**A**H	**A/**A	**A**H/**A**
Goblet cells	**B**/**B**H	**AH**	**A**/A/**A**H	**A**H/**AB**H
LARGE INTESTINE (1; 2; 2; 1)				
Crypts	**B**	**AH**/**A**H	**A**H/BH	**AB**H
Goblet cells	**B**	**AH**/**A**H	**A**H/**BH**	**AB**H
PANCREAS (1; 0; 2; 0; 2)		NT	n = 2	NT
Islets	B	-	A	-
Acini	B	-	A	-
Ducts	**B**	-	A	-
BLADDER (2; 4; 1; 4)				
Surface cells	NE	**A**/A*	A	SE/A*
Intermediates	**B**/**B**H	**A**/A*	A	**A**/**AB**
Basal	B/**B**H	**A**/A*	A	**A/A**B/AB**H**
SALIVARY GLAND (1; 2; 1; 3)				
Ducts	**B**	**A**/A	BH	A*/AB
Myoepithelial cells	NE	NE	NE	**NE**
Serous glands	NE	A	**B**H	AH/AB/**AB**
Mucosal glands	**B**H	**A**H/**A**	**BH**	**A/ABH/AB**H
LIVER (2; 6; 6; 4)				
Hepatocytes	NE	NE	NE	NE
Sinusoidal endothelium	NE	NE	NE	NE
Bile canaliculi	NE	NE	NE	NE
Greater ducts	**B**	AH/A*H^S^	**A**	A/AB*/AB^S^H
Lesser ducts	**B/**B	**A**H/AH/AH^S^	**A/**B	A/AB*/AB^S^
THYROID	NT	n = 1	n = 1	NT
Follicular cells	-	NE	NE	-
Parafollicular cells	-	NE	NE	-
VAGINA (2; 2; 2; 2)				
Surface cells	**B**/NE	**AH**/**A**H	**AH**	**AH/AB**
Intermediate	**B**/B	**A**H	**A/AH**	**AH**/**AB**H
Basal	**B**/B	**A**H	**A**	**H**/H
PROSTATE (0; 1; 1; 0)				
Glandular epithelium	-	A	**A**	-

/ = or; NT = Not tested; NE = No expression;

* = related to one animal;

Lack of bold type = Heterogeneous expression pattern of the A antigen analyzed; Bold type = Homogeneous expression pattern of the A antigen analyzed;

^**S**^ = Surface expression.

**Table 3:** Expression of ABH antigens in organs of mesodermal origin in species of New World Monkeys.

TISSUE Layer/ (n) cell type	Species/ABH Expression			
	*A*. *infulatus*	*C*. *jacchus*	*S*. *apella*	*S*. *sciureus*
UTERUS (2; 3; 2; 1)				
Endometrium	B/BH	H*/A**H**	**A**/**A**H	**AB**
UTERINE TUBE (2; 3; 3; 2)				
Ampulla and Fimbria	**B/B**H	AH*/**H**	A**H**/H*	**A/AB**
OVARY (1; 3; 3; 2)				
Ovarian epithelium	B	NE/A*	A/**A**/**A**H	**A**
TESTICLES/RELATED STRUCTURES (0; 1; 2; 0)				
Vas deferens	-	NE	**NE/BH**	-
Efferent duct	-	A**H**	A/B	-
Epididymis	-	A	NE	-
Testicle	-	NE	NE	-
Epithelium of the seminal vesicle	-	A	AH/BH	-
SPLEEN (3; 1; 6; 2)				
White Pulp	NE	NE	NE/A*	NE/BH
Red Pulp	NE	NE	NE	NE/ABH
KIDNEY (0; 5; 2; 3)				
Glomerular epithelium	-	NE	NE	NE
Proximal tubules	-	NE	NE	NE
Loop of Henle	-	NE*/A	A/B	A/**A**B/**AB**
Distal tubules	-	NE*/A	A/B	A/AB/NE
Collecting Ducts	-	A/A*	A/B	A/**A**B/AB
Collecting Tubules	-	A	A/B	A/AB/ABH

/ = or; NT = Not tested; NE = No expression;

* = related to one animal;

Lack of bold type = Heterogeneous expression pattern of the A antigen analyzed; Bold type = Homogeneous expression pattern of the A antigen analyzed; ^**S**^ = Surface expression.

#### Aotus infulatus

All of the animals are monomorphic belonging to the human B type group. One study [19] found that *A*. *infulatus* expressed only B phenotype. Table 1 lists the pattern for expression of antigens B and H depending on the type of cell or tissue layer. For example, only B antigen was detected in the basal layer of the esophagus (Fig 1a), while substance H was also expressed in the gastric mucosa (Fig 1b), in goblet cells of the small intestine (Fig 1c), and in intermediary and basal cells of the epithelium in the bladder (Fig 1d).

#### Callithrix jacchus

In this species, only animals of group A human blood type were found. In *C*. *jacchus* the ABO system was monomorphic, where all individuals were classified as A phenotype, as reported by Rocha et al. (1990) [19] and Schneider *et al*. (1985) [20]. The A antigen was expressed in some areas such as the loop of Henle in renal tissue (Fig 1e), in the prostate (Fig 1f), in the seminal vesicles and spermatozoids (Fig 1g). H antigen was also expressed in the hepatic tissue (Fig 1h).

#### Sapajus paella

Animals were found with phenotypes of human blood groups type A and type B. But, in this species, all four phenotypes (A, B, AB, O) have been found as described in Schneider *et al*. (1985) [20] and Corvelo *et al*. (2002) [21]. The H antigen was in basal cells of the pharyngeal epithelium (Fig 2a) and in the ducts, mucosal and serous acini of the salivary gland (Fig 2b). The expression of the A antigen in the prostatic epithelium is demonstrated in Fig 2c while the expression of B antigen is demonstrated in Fig 2d in the small intestine, especially in goblet cells.

#### Saimiri sciureus

In this species, human-type A and AB types were found. However, the squirrel monkeys were observed to have all four phenotypes [22, 23]. The expression of H antigen is identified in the basal layer of the vaginal epithelium (Fig 2e) and bladder epithelium (Fig 2f). The B antigen may be found in the major and minor hepatic ducts in the phenotype AB animal (Fig 2g) and in all layers of the gastric mucosa (Fig 2h). Only the B antigen may be detected in the white pulp of the spleen in an individual of phenotype AB.

**Fig 3:**
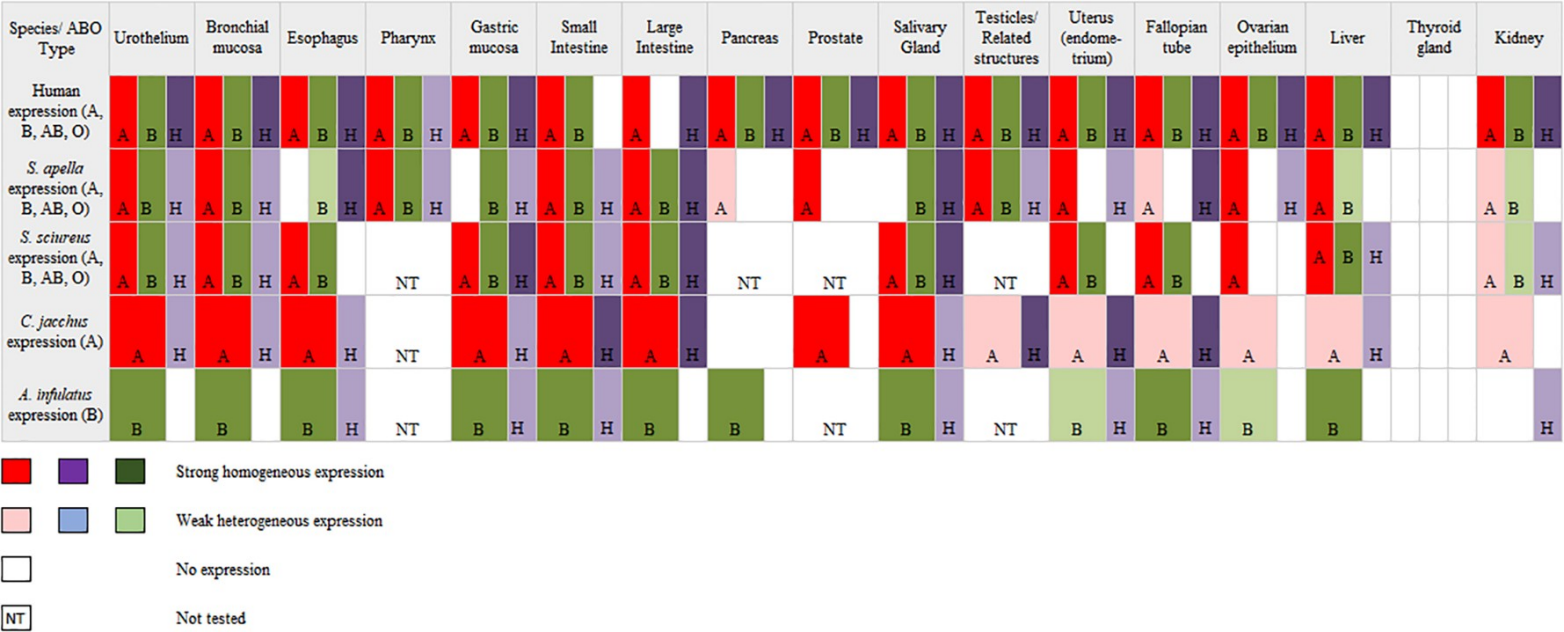
Schematic drawing of comparison of ABH histoblood group expression in several tissues from New World Monkey and human.

*Intra-and interspecific diversity of the ABH expression in NWM*. As can be seen in Table 4, compatible ABH expression was verified in all animals examined. Based on score ABH from histological expression distributions in both origin tissues (endo-/mesodermal), the occurrence of maximum diversity revealed more heterogeneity of H antigens than A/B antigens, which seemed to show homogeneity (Fig 3). Moreover, in some of the different cell/tissue layers of endo- and mesodermal organs were found more diversity to B antigen expression in *A*. *infulatus* than *S*. *sciureus* and *S*. *apella*. Nevertheless, in selection to A antigen, the higher estimated diversity indexes divide the analyzed species in *C*. *jacchus* from *S*. *sciureus* and *S*. *apella*.

**Table 4:** Histological expression score of ABH antigens in organs of endo-and mesodermal origin in New World Monkey species.

Species	Organs origin	Histological score of ABH antigens																	
		A						B						H					
		+++	++	Total*	μ ±	SD	ID	+++	++	Total*	μ ±	SD	ID	+++	++	Total*	μ ±	SD	ID
*S sciureus*	Mesodermal	12	6	18	2.67	0.48		4	2	6	2.67	0.52		-	3	3	2.00	0	
	Endodermal	19	1	20	2.95	0.22		9	3	12	2.75	0.45		3	6	9	2.25	0.45	
							1.58						1.25						1.07
*A*. *infulatus*	Mesodermal	-	-	-	-	-		7	5	12	2.58	0.51		-	4	4	2.00	0	
	Endodermal	-	-	-	-	-		12	3	15	2.80	0.51		1	6	7	2.14	0.38	
																			1.04
*S*. *apella*	Mesodermal	6	9	15	2.40	0.51		1	4	5	2.20	0.45		2	4	6	2.33	0.52	
	Endodermal	13	5	18	2.72	0.46		6	2	8	2.75	0.46		5	6	11	2.45	0.52	
							1.51						1.11						1.22
*C*. *jacchus*	Mesodermal	11	8	19	2.58	0.51		-	-	-	-	-		8	6	14	2.57	0.51	
	Endodermal	14	8	22	2.64	0.49		-	-	-	-	-		6	6	12	2.50	0.52	
							1.60						1.42						1.41

*Tissue specimes; SD = Standart Deviation; ID = Shannon-Wiener diversity index.

In this way, the statistical analysis by (Shannon-Wiener Index) showed that the diversity is confirmed by a scenario, in which the history of the species is result of differences in selection pressures among these closely related species so that the variation at ABO system reflects a multiallelic balanced polymorphism.

## Discussion

In this study, the tissue distribution pattern of human ABH type in NWP was similar to patterns of expression in humans. In that regard, the results analyzed related to the expression of this histo-antigens in tissue structures of endodermal and mesodermal origin in the species *S*. *sciureus*, *A*. *infulatus*, *S*. *apella* and *C*. *jacchus*, revealed some findings regarding the reactivity of these ABH antigens, defining distinct patterns of antigen expression because of this intra and interspecies diversity.

In a comparison of ABO status and ABH immunohistological expression antigens of NWM species and human were observed that these distributions seemed to be dependent on the cell/tissue type, where these ABH antigens are formed by the sequential addition of monosaccharides to the growing chain in relations to cellular differentiation as proposed by Ravn and Dabelsteen (2000) [3]. Our findings are also consistent with their hypothesis. However, some changes into the ABH patterns of the expression antigens were found in various cell/tissue layers of the NWM species. A likely mechanism for these differences may be due to the regulation of the types of precursor carbohydrate chains and the activity of glycosyltransferase enzymes (*FUT1*; *FUT2*; *Sec1*; *A/B* genes) involved in the synthesis of A/B/H antigens (Fig 3).

In the overall framework of this study, for each ABH antigen, there seems to be a pattern of tissue expression, conserved and restricted to species, probably because of differentiation in the tissue organization. This results from gene rearrangements and polymorphisms with antigenic activity during morphogenesis. Because of this biochemical diversity, one may view the ABH antigens preferentially as immunological macromolecules, which determine an active role at the cell surface, and thus participate in cell migration and mutual recognition, which are indispensable for biological processes.

In this study, comparison of the results was done with the assumption that the genetic pattern of the expression of these ABH type human carbohydrate antigens. These antigens would exhibit the same type of genetic control found in humans, marked by preferential use of the type 1 chain by the human-type FUT2 enzyme (*Se*), in tissues with endodermal origin, and by action of the human-type FUT1 enzyme (*H*) in the type 2 chain, in tissues with an ectodermal and mesodermal origin [15]. Furthermore, in this interpretation, one should note the data from analyses of the sequences in *Sec1* in primates [16], which supposedly also codify for a potentially functional α-2-fucosiltransferase in OWM and NWM, although it is a pseudogene in humans. Those assumptions were adopted to inform these first studies and identify the probable divergences between these non-human primates and humans. From there, one may better understand how these differences in ABH expression depend upon an evolutionary pattern of gene regulation.

Investigation of the expression of ABH antigens in primate tissues is restricted to a handful of studies. These demonstrate expression in epithelial tissues, endothelium and secretions in new and OWP, using antibodies and lectins [13–15], without, however, a detailed description of reactive cell types in the tissue of each of the species studied, as has been done exhaustively with humans [3]. Expression of these antigens in tissues is related to the embryonic origin, cell differentiation and gene control in the H and Se systems of the tissues involved [11, 12].

In this study, with regard to the wide distribution of these antigens, the expression of the H antigen showed great diversity, particularly of the intra-specific type. This expressed variation is because this H antigen serves as a precursor for production of the A and B antigens. Furthermore, one cannot ignore that the distinct expression patterns of substance H are the result of the type of gene regulation, which may be dependent on the synthesis of three distinct α-2-fucosiltransferases that are the product of genes *H*, *Se* and *Sec1* described in NWM [6]. Another aspect that must be emphasized are the discrepancies observed in comparative patterns between humans and NWM, in different tissue structures with distinct embryonic origins. The stratified epitheliums of the esophagus and pharynx demonstrated a heterogeneous expression of at least one of the ABH antigens in the basal layer. This observation is different from what has been reported humans [24, 25], where only the expression of the H precursor was found in a few parabasal cells [26]. Considering the constant cell renewal in those epitheliums, the basal layer, with a view to cellular differentiation, accordingly modulates the synthesis of new antigenic ABH structures on the cell surface, which reinforces this differentiation in the antigenic intra and interspecies expression.

In the gastric mucosa, it was found that the difference in the expression of the H antigen, when compared with data from humans, was related to antigen distribution, with more homogeneity in tissue expression found in humans [27, 28]. Although Ravn and Dabelsteen (2000) [3] did not indicate the presence of the antigen in cells small intestine cells in humans, Shimamoto *et al*. in 1987 [29] revealed reduced and rare expression patterns for the H antigen. In this study, the results found for the H antigen were of a more intense marking in goblet cells than that reported for humans by Shimamoto *et al*. (1987) [29].

Studies performed in human large intestines describe ABH antigens being found in absorbent and goblet cells, with a tendency towards higher expression in goblet cells similar to what was observed in the species analyzed in this study [24, 25, 30, 31].

In the bladder epithelium, the detection of ABH antigens differed between the primate species studied, with some species having a reduction and/or absence of expression. In terms of humans, expression of these antigens at the surface layer also showed variation with loss or reduction in antigen expression [24, 25, 32, 33] as described above for the monkeys investigated. This variation observed in surface layer cells in the bladder may be a sign of a process of cell aging (desquamation) that leads to the concurrent disappearance of antigens.

In all the species studied, expression of the H antigen was in most cases detected in the mucous glands of the salivary glands. While in the ducts and serous glands, this pattern varied considerably between the species. It may be due to the chemical nature of the ABH substances produced by the different glands, where mucous glands have a production rich in glycoproteins, while in serous glands their concentration is more diluted, making detection of those antigens more difficult [34].

In the animals’ livers antigen marking was found in the major and minor ducts. In humans, studies indicate that marking is not found in the minor ducts [34]. Therefore, those found positive ABH marking in both ducts apparently are reproducing a distribution pattern found in the fetal and post-natal phase of development observed in humans, which in the case of the primates seems to have persisted in both structures in the organs until the adult phase [35].

In the follicular cells of the thyroid gland of the species analyzed, there was no expression of ABH antigens, as also described for humans [3, 24, 25]. As for the vaginal epithelium, the ABH expression pattern is different from humans, where the expression occur in the upper layer with a tendency towards reduction of expression in the intermediate layer and absence in the basal layer [24, 25]. Among the studied monkeys, many basal cells in the vaginal epithelium are capable of expressing ABH already at that level of differentiation. These findings thus seem to demonstrate that this profile of expression in the basal layer results from the ontogenetic process, as described for hepatic ducts.

In the prostatic epithelium, there was no expression of the H antigen in the studied monkey species. In humans, descriptions of the presence of H antigen in this epithelium differ among some of the studies. One study [36] did not find expression of this antigen, unlike what was cited in the review by Ravn and Dabelsteen, 2000 [3]. It is thus very likely that non-detection of the H antigen may be a common event, given that this substance is a precursor for antigens in the ABO *locus*. In the human uterine endometrium and uterine tube, hormonal influences control the growth, differentiation and expression of ABH antigens. The increase in expression of these antigens occurs mainly in the late and secretory proliferative phases [37]. In the study, the female monkeys were in intermediate and late stages of the proliferative phase in the reproductive cycle. The endometrium and uterine tube expressed the ABH antigens in a very similar manner among the species studied, and in humans [3]. Welshinger *et al*. in 1996 [38] note that in humans, ABH antigens are normally not found in the ovarian epithelium. On the other hand, when there is hormonal activation in this epithelium, a change in the cellular morphology is induced with synthesis of new proteins, including ABH antigens, as found in this analysis.

In males, the testicles and related structure examined in *Callithrix* and *Sapajus* showed differences in detection of ABH antigens between the species. Thus, a larger sampling number was necessary in order to establish a pattern for ABH expression. However, *C*. *jacchus* was the species presenting the greatest similarity in relation to the marking profile described for these humans [39].

In human spleen cells, there is no presence of ABH antigens [24, 25]. This pattern was also reproduced in the majority of the monkeys tested. However, two animals species were found *Sapajus* and *Saimiri*, with reactivity for the A and BH antigens, respectively in the white pulp, and for A, B and H in the red pulp *Saimiri*. This shows a distinct cell-specific pattern type in those areas. Although one may point out that, no histological modification was observed that justify this change in the pattern for histological expression. The appearance of these molecules may be the result of a positive cellular regulation for antigenic activity and the persistence of this phylogenetic level [40].

Human renal tissue shows a permanent expression of ABH antigens at the level of collecting and distal ducts and tubules, but not in the loop of Henle [41, 42]. In the primates studied, the detection of these antigens was also observed in this structure in 98% of the animals. That characteristic indicates a differentiated antigenic expression in this structure between members of the families studied and humans.

It was found that differences in the expression of ABH antigens between New World monkey species and humans. This occur mainly in the structures of organs that have undergone ontogenetic, morphological and functional differences. Thus, constant antigenic reactivity was detected on many epithelial cells of all the tissues and organs, which have secretory functions. Thus, they are under apparent control of the like-human FUT-2 (*Se*) enzyme. On the other hand, a tendency towards the absence or reduction of A/B antigens, and particularly of the H precursor substance was a typical finding in organs of mesodermal origin that apparently are under the control of the like-human FUT-1 (*H*) enzyme. It is thus plausible that specific predictions regarding the functions of these ABH blood group antigens may be derived from the results of analyses of consistent species-specific variations in the tissue distribution of these antigens. This tissue distribution with reactivity patterns that are similar and conserved between the different species, reinforce the evidence of the evolutionary impact of ABH antigens.

The ABO glcosyltransferase and α-2-fucosyltransferase families’ genes are polymorphic at least in humans. Although in some primates species these genes are monomorphic or show low-frequency polymorphism (i.g. *C*. *jacchus* and *A*. *infulatus*). In this case, is expected that the genic product act at the cellular level where they are indispensable in cell-cell interactions, occurring during differentiation, organogenesis and in the inflammatory process, then these genes are essential and so conserved among the species. In contrast, the multiantigenic variations (or intraspecies diversity) are mainly present on epithelial cells in contact with the external environment. The biological meaning is that various microorganism bind to the epithelial cells via glicoconjugate, expressing either precursor of ABH antigens (non-secretor) or the ABH antigens themselves (secretor). These polymorphisms might also split the population between sensitive and resistant individuals to diseases, due to difference in selection pressures among the species. Furthermore, this study help to understand that the ABH antigens are the result of an ancient polymorphism, in which some alleles are shared among related species (such as *S*. *apella* and *S*. *sciureus*) and maintained under balancing selection.
