# Correction: Tissue expression of antigens of ABH blood groups in species of New World Monkeys (*Aotus infulatus*, *Callithrix jacchus*, *Sapajus apella and Saimiri sciureus*)

**DOI:** 10.1371/journal.pone.0259504

**Published:** 2021-10-28

**Authors:** Délia Cristina Figueira Aguiar, Washington Luiz Assunção Pereira, Gyselly de Cássia Bastos de Matos, Klena Sarges Marruaz da Silva, Rosane do Socorro Pompeu de Loiola, Tereza Cristina Oliveira Corvelo

There is an error in Fig 3. The authors have provided a corrected version here.

**Fig 3:**
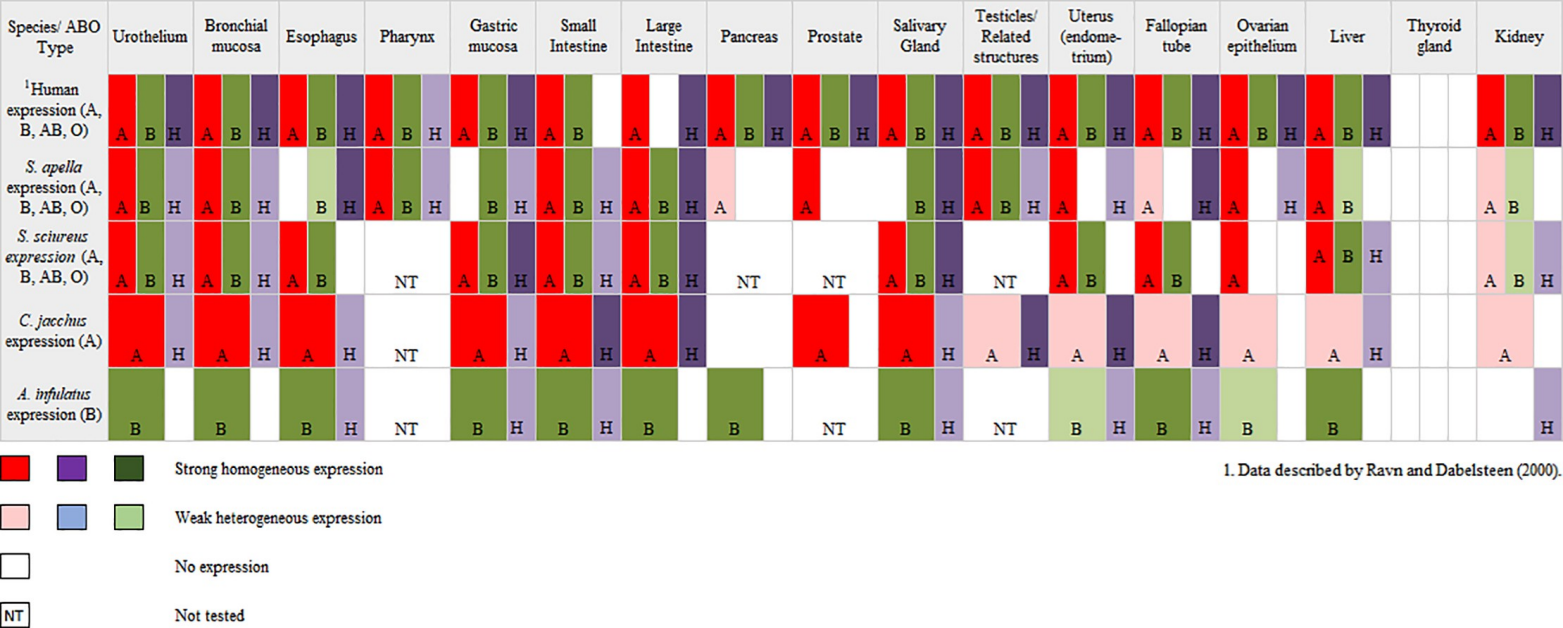
Schematic drawing of comparison of ABH histoblood group expression in several tissues from New World Monkey and human.
